# Mitochondrial roles in disease: a box full of surprises

**DOI:** 10.15252/emmm.201505350

**Published:** 2015-07-20

**Authors:** Anu Suomalainen

**Affiliations:** 1 Research Programs Unit, Molecular NeurologyHelsinki, Finland; 2 Neuroscience Center, University of HelsinkiHelsinki, Finland; 3 Department of Neurology, Helsinki University Central HospitalHelsinki, Finland

## Abstract

This commentary inaugurates a new review series in *EMBO Molecular Medicine* focused on mitochondrial diseases. This area of medicine, which actually encompasses most disease areas, has long since come of age and is now positioned for the next leap toward the development of effective therapies. The aims of the review series are to offer a comprehensive overview of this exciting area of medicine and research and to provide timely discussions for clinicians and investigators on the new discoveries elucidating how mitochondrial metabolism contributes to an expanding group of complex, heterogeneous, and difficult-to-tackle diseases.

In 1962, Rolf Luft reported on the first patient suffering from a hypermetabolic disorder due to mitochondrial dysfunction (Luft *et al*, 1962). In the 1970s and 1980s, the basic functional components of human mitochondrial biology were established, including mitochondrial DNA sequence, transcription machinery, and respiratory chain biochemistry. However, the field ignited in 1988 with the first description of mtDNA mutations in human disease (Holt *et al*, 1988; Wallace *et al*, 1988). During the 1990s, mtDNA genetics was established as an important aspect of human medicine and the first nuclear gene defects underlying mitochondrial dysfunction were found (Bourgeron *et al*, 1995). Over 1,200 nuclear genes encode proteins targeted to mitochondria, and even more proteins are somehow functionally linked with the organelle. The fact that > 6% of the active genome is dedicated to the maintenance and function of mitochondria *per se* emphasizes the crucial role of the mitochondria: transforming nutrient energy to ATP and fine-tuning cellular growth and catabolism in response to the environmental and physiological demands (Nunnari & Suomalainen, 2012).

Only two Luft disease patients have been reported to date, exemplifying a typical aspect of mitochondrial disease: a constellation of hundreds of individual rare disorders with various and differing clinical signs. The patients often manifest with neurological symptoms, but may also present with cardiomyopathy, sudden blindness, hearing loss or psychiatric symptoms—almost any symptom really—making the disorders especially difficult to diagnose. Mitochondrial diseases are the most common causes of inherited metabolic disease in adults and children and yet are still relatively poorly known among medical practitioners. Indeed, with an overall frequency of ∼;1:2,000, almost 400,000 people in Europe suffer from a classic genetic mitochondrial disease. National and international patient organizations, such as International Mitopatients and United Mitochondrial Disease Foundation, do invaluable work in raising awareness and providing information for health practitioners, patients, and their families.

During the past decade, the amount of knowledge on nuclear genetic causes of disorders with mitochondrial dysfunction has extensively increased, providing us with tools to classify diseases based on genetic background. This has enabled international natural history studies and treatment trials for the rare disorders based on genetic and phenotypic homogeneity. The genetic knowledge has also uncovered phenotypic mimicry, for example, between mitochondrial disorders and vitamin metabolic defects as vitamin B1 and B9 transporter deficiencies (SLC19A3; FolR1), which manifest with mitochondrial disease-like clinical symptoms (Leigh-syndrome-like, Alpers-syndrome-like, respectively). These defects lead to progressive, lethal neurological disorders of childhood, if untreated, but early vitamin supplementation can prevent progression or even the manifestation of severe symptoms. The knowledge that folate- and thiamin transporter defects are partially curable emphasizes first-line genetic testing and empirical vitamin B supplementation to infants immediately upon suspicion of mitochondrial encephalopathy, even before establishing a molecular diagnosis. The number of treatable mitochondrial or mitochondrial-like disorders is slowly growing, along with the increased availability of diagnostic tools and knowledge of the pathogenic mechanisms. Most mitochondrial diseases, unfortunately, remain without any cure.

Molecular genetics discoveries have enabled the generation of valuable disease models, which have dramatically increased our understanding of early disease physiology and provided completely new vistas on molecular pathogenesis and possible avenues of treatment. Specifically, the accrued knowledge indicates four modes through which mitochondria contribute to health and disease: (i) intramitochondrial catabolic mechanisms of oxidative phosphorylation; (ii) mitochondrial signals and metabolites that affect cytoplasmic organellar functions (autophagy, endoplasmic reticulum, lysosomes, peroxisomes) and intermediary metabolism; (iii) mitochondrial roles in inducing metabolic cytokine secretion and tuning distant organs; and (iv) genetic susceptibility of mitochondria to external insults. These mechanisms have high relevance for understanding both the unique spectrum of tissue-specific mitochondrial disease manifestations and mechanisms of cellular maintenance and degenerative diseases, including neurodegeneration, stem cell biology, and aging.

The series begins with Valeria Tiranti's review in this issue on the genetic defects exposing mitochondria to environmental/metabolic toxins. Tiranti *et al* originally discovered *ETHE1* mutations as causative of an atypical mitochondrial disease clinical phenotype, involving microangiopathy, diarrhea, and progressive early-onset encephalopathy, associated with mitochondrial cytochrome c oxidase (COX) deficiency. *ETHE1* encodes a persulfide oxidase that neutralizes hydrogen sulfide, a COX inhibitor and gasotransmitter. Ethe1 deficiency in patients leads to increased H_2_S levels in tissues, as well as in the intestine, where anaerobic bacteria produce the gas. Remarkably, diarrhea was relieved by the antibiotic metronidazole and microangiopathy by N-acetyl-cysteine, a glutathione precursor (Tiranti *et al*, 2009). These findings introduced completely new concepts in mitochondrial medicine, by indicating the susceptibility of intra-organellar metabolism to changes in the cytoplasmic and even extracellular environments. Furthermore, they indicated that a genetic defect can sensitize intestinal cell mitochondria to metabolites produced by gut bacteria, leading to clinical manifestations. Understanding basic intermediary metabolism and its links to mitochondrial function and disease may well reveal similar pathogenic routes underlying progressive disorders.

Mitochondria are the organelles that burn nutrients to produce the main energy currency, ATP. It should therefore come to no surprise that their dysfunction impacts nutrient signaling. Recent data obtained in mitochondrial disease mouse models have indicated that modifying NAD^+^ metabolism for example with vitamin B3 derivatives may delay progression of adult-onset mitochondrial myopathy (Khan *et al*, 2014), leading to mitochondrial biogenesis and activation of sirtuins, thus linking late-onset mitochondrial disease pathogenesis directly with abnormal nutrient sensing mechanisms. Furthermore, mitochondrial myopathy induces the secretion of a metabolic cytokine, FGF21, from muscle to blood, recruiting lipids from fat stores (Tyynismaa *et al*, 2010). This indicates that a single tissue with mitochondrial dysfunction can have direct metabolic consequences in the whole organism in a cell non-autonomous fashion. In addition to its mechanistic relevance, FGF21 has also become a useful serum marker for mitochondrial muscle disease. The mechanistic implications of these findings are also relevant for conditions with secondary mitochondrial dysfunction, including aging. I will discuss the impact of mitochondrial dysfunction in nutrient signaling and the therapeutic opportunities offered by this knowledge in another upcoming review.

Mitochondria are dynamic organelles, which change their shape and location depending on cellular cues and needs, in tight interaction with the cytoskeleton and cellular events such as calcium currents in neurons (Kasahara & Scorrano, 2014). Furthermore, mitochondria interact with other cellular organelles, which in turn regulate their dynamics and metabolism. For example, the endoplasmic reticulum has direct tight contacts with mitochondria, marking the sites of organelle division (Friedman *et al*, 2011), and mitochondria can bud small vesicles fusing to peroxisomes or lysosomes and carrying cargo (Soubannier *et al*, 2012). The potential of treatment strategies affecting mitochondrial division machinery, morphology, recycling and repair are an active field of research, and will be the topic of Luca Scorrano's contribution to this series.

Mitochondrial signals can modify cellular behavior and differentiation at multiple levels. Despite the assumption that mitochondrial dysfunction and aging are associated with increased amounts of reactive oxygen species (ROS), evidence of increased oxidative damage has not been found in mouse models with mtDNA mutagenesis (Trifunovic *et al*, 2004; Kujoth *et al*, 2005). Instead, these models have indicated the sensitivity of the stem cell compartment to subtle increases in superoxide radicals, which commit progenitor cells toward proliferation and differentiation (Ahlqvist *et al*, 2012; Hämäläinen *et al*, 2015). Indeed, mitochondrial quiescence is essential for stem cell quiescence; mitochondrial mutagenesis in pluripotent tissue stem cells is thus a threat for maintenance of their stemness. The importance of ROS signaling for stem cells makes them also sensitive to mitochondrial antioxidants—a completely understudied field, and an important aspect in assessing intervention effects for different cell types. Mike Murphy will discuss the exciting field of ROS biology in mitochondrial disease in another upcoming review.

Finally, two reviews by Patrick Chinnery and Jan Smeitink will present and discuss the complex, complementary, and yet diverging clinical and genetic landscapes of adult and pediatric mitochondrial disease, respectively.

Nutrients, vitamins, metabolites, and antioxidants can crucially affect mitochondrial homeostasis and cell maintenance. Understanding the molecular mechanisms of the cross talk between mitochondria and the cellular and extracellular environments in different tissues will be the key to explaining the variable manifestations of mitochondrial pathology, assessing treatment options and their adverse effects. Considerable recent increase in knowledge of mitochondrial disease physiology in mice, however, justifies careful optimism concerning treatment of mitochondrial dysfunction and challenges scientists to take the essential steps toward human trials.
